# Supplementary material for: Mid-Miocene warmth pushed fossil coral calcification to physiological limits in high-latitude reefs
Source: Commun Earth Environ. 2025 Jul 19;6(1):569. doi: 10.1038/s43247-025-02559-9 (PMC12274133; doi:10.1038/s43247-025-02559-9)
Supplement: Supplementary file 2 — Supplementary Information [file 43247_2025_2559_MOESM2_ESM.pdf]

Supplementary Information for

Mid-Miocene warmth pushed fossil coral calcification to physiological limits in high-latitude reefs

Markus Reuter[1*], Juan P. D'Olivo[2], Thomas C. Brachert[3], Philipp M. Spreter[3], Regina Mertz-Kraus[4] & Claudia Wrozyna[1]

[1]Institute of Geography and Geology, University of Greifswald, Friedrich-Ludwig-Jahn-Straße 17a, 17489 Greifswald, Germany

[2]Reef Systems Academic Unit, National Autonomous University of Mexico (UNAM), Prol. Av. Niños Héroes S/N, 77580 Puerto Morelos, Quintana Roo, Mexico

[3]Institute for Earth System Science and Remote Sensing, Leipzig University, Talstraße 35, 04103 Leipzig, Germany

[4]Institute of Geosciences, Johannes Gutenberg University Mainz, Johann-Joachim-Becher-Weg 21, 55128 Mainz, Germany

[*]Corresponding author: markus.reuter@uni-greifswald.de

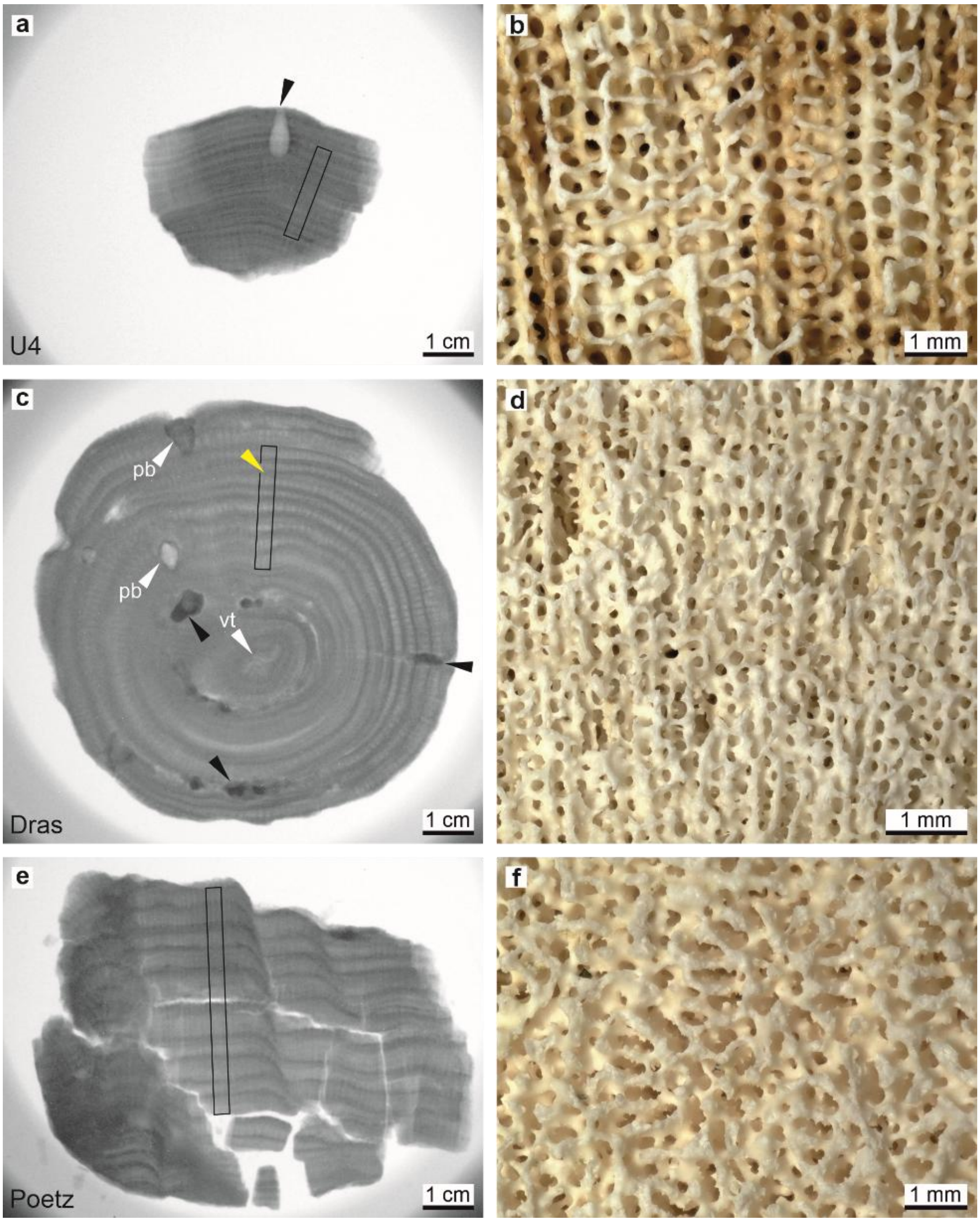

Supplementary Figure 1: X-ray images and skeletal details illustrating the preservation of the fossil *Porites*. **a** X-ray positive of U4 showing annual density banding. The black arrow points to a bivalve boring. The black box marks the measurement transect for density and geochemical records; applies also for (**c**) and (**d**). **b** Reflected light microscope image of U4, longitudinal section of corallites. In contrast to samples Dras and Poetz, the skeleton of U4 exhibits brown-colored areas due to very thin iron coatings. As the white and brown areas cannot be distinguished on the X-ray image, the iron coatings were found to be volumetrically insignificant for determination of skeletal density. **c** X-ray positive of Dras depicting skeletal density banding. The *Porites* corallith developed around a vermetid tube (vt) and was

inhabited by pyrgomatid barnacles (pb). The high-density band between years 6 and 7 in the coral proxy record (indicated by a yellow arrow in the density measurement transect) represents a growth hiatus. The hiatus is laterally equivalent to an annual growth increment that become thinner and pinch out laterally. This stratal pattern is interpreted to reflect rotation of the mobile corallith. The black arrows indicate skeletal breakouts that were filled during sample preparation with dental composite resin (Estelite Universal Flow) for stabilization. **d** Reflected light microscope image of Dras, longitudinal section of corallites. **e** X-ray positive of Poetz recording the skeletal growth banding. **f** Reflected light microscope image of Poetz, transverse section of corallites.

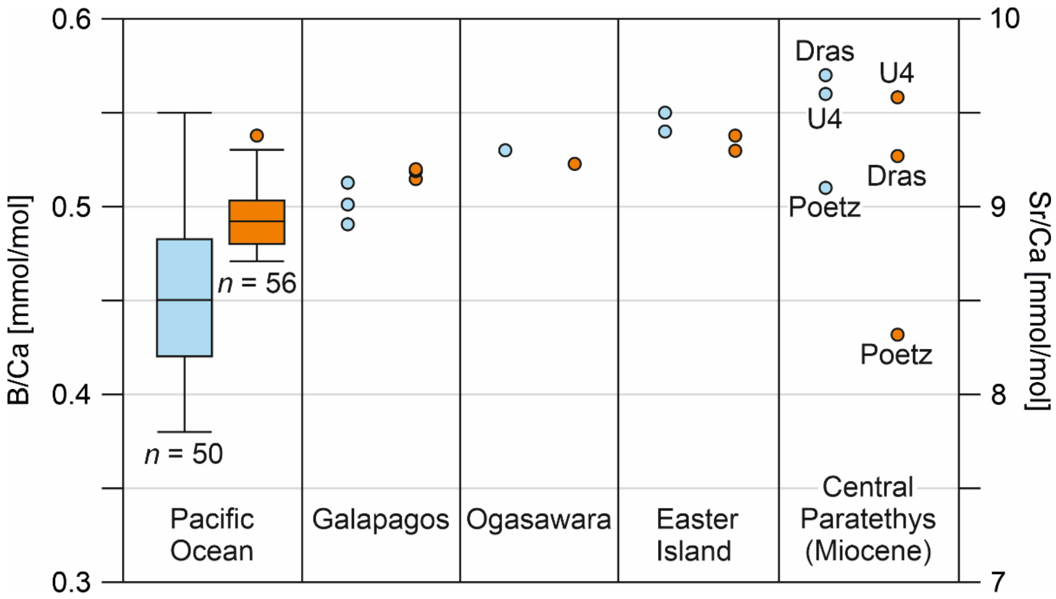

Supplementary Figure 2: Comparison of Sr/Ca (orange) and B/Ca (light blue) mean values between mid-Miocene *Porites* corals from the Central Paratethys (U4, Dras, Poetz) and modern *Porites* corals from the Pacific[1–5]. The B/Ca values of U4 and Dras as well as the Sr/Ca value of U4 exceed the range of modern corals. The B/Ca value of Poetz and the Sr/Ca value of Dras are above the interquartile range of modern *Porites* in the range of corals from extreme and marginal habitats. Remarkably, the Sr/Ca value of Poetz is far below the values of U4 and Dras, and the range of modern corals. This deviation is probably due to the strong intra-annual variations in linear extension rate, which result in warm seasons with high extension rate but low Sr/Ca (and low B/Ca) values (Fig. 3c) being disproportionately included in the coral mean value.

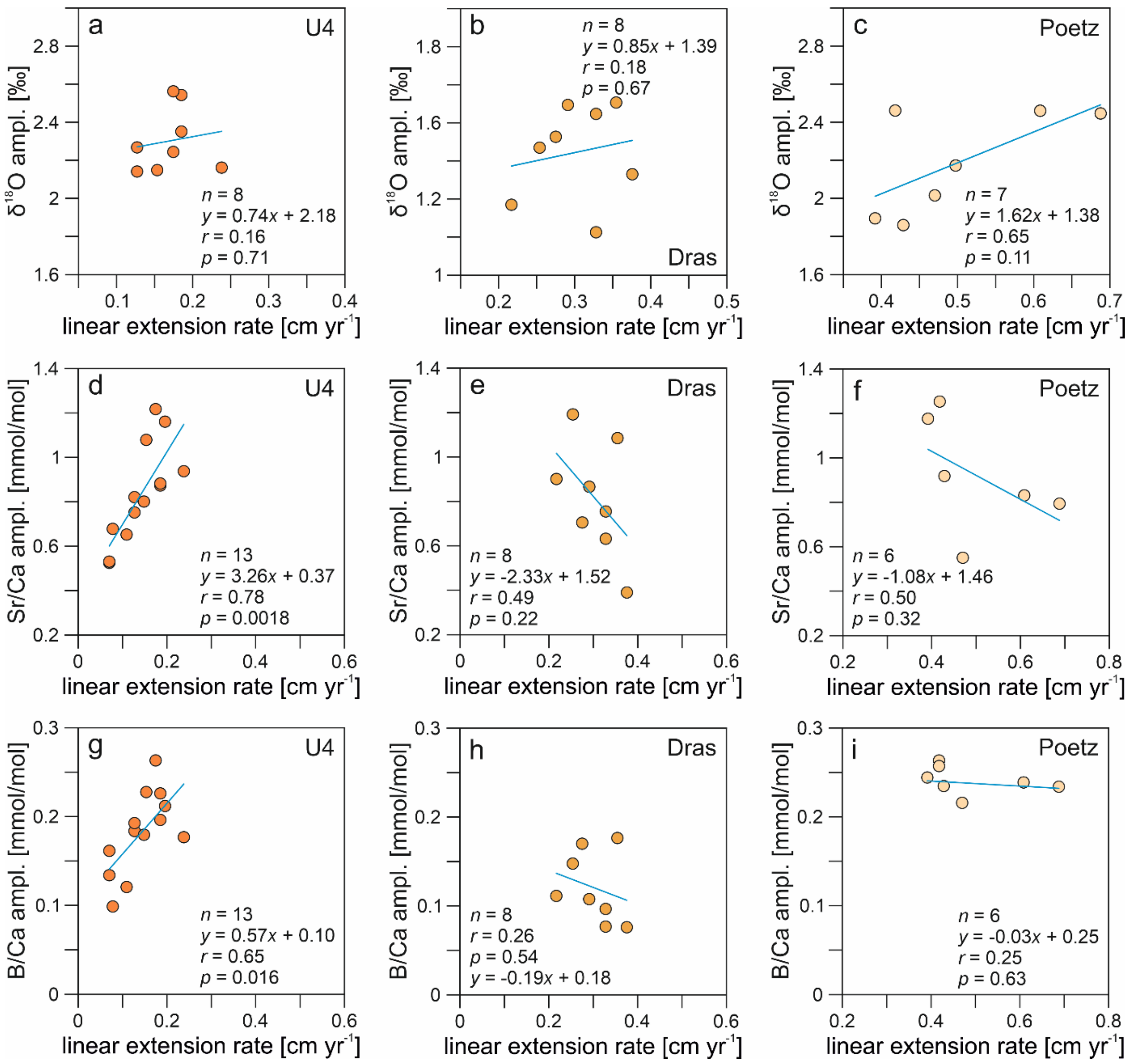

Supplementary Figure 3: Relationship between linear extension rate and amplitude of coral proxy annual cycles. **a–c** δ^{18}O. **d–f** Sr/Ca (5-point moving averages). **g–I** B/Ca (5-point moving averages).

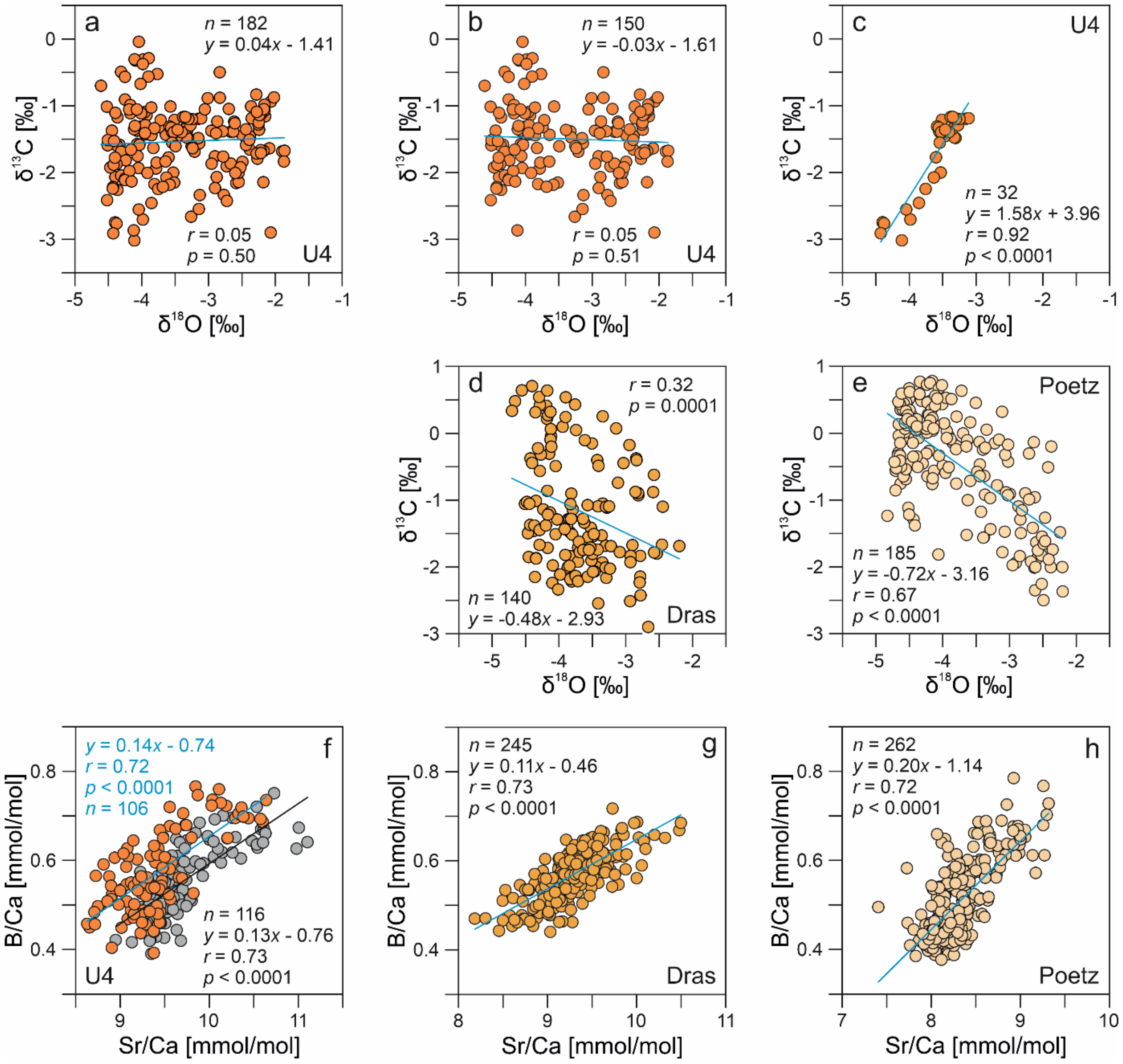

Supplementary Figure 4: Coral $\delta^{13}C$ vs. $\delta^{18}O$ (**a–e**) and B/Ca vs. Sr/Ca (**f–h**) cross-plots for Middle Miocene *Porites*. **a** Scatter plot of the complete stable isotope dataset for U4. **b** $\delta^{13}C$–$\delta^{18}O$ relationship in undisturbed $\delta^{18}O$ cycles of U4. **c** Scatter plot of stable isotope data in the post-disturbance interval of U4, characterized by the loss of $\delta^{18}O$ cyclicity. **f** Sr/Ca baseline shift in U4. Data from before the disturbance event are orange, and data from this event onwards are grey.

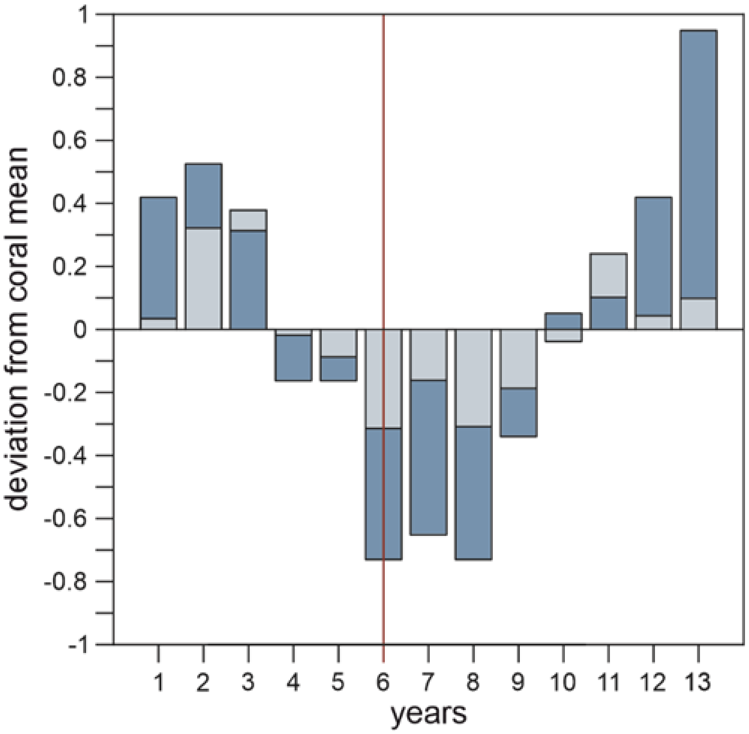

Supplementary Figure 5: Growth anomaly in the early Langhian/early Badenian *Porites* (U4) based on Sr/Ca. The dark bars show the deviation of the annual linear extension rate (cm yr^{-1}) from the coral mean (all years) and the light bars the deviation of the annual Sr/Ca amplitude height (mmol/mol). The vertical red line indicates the timing of the inferred bleaching event.

Supplementary Table 1: Analytical results of LA-ICP-MS measurements for the quality control materials together with reference values.

Element	USGS BCR-2G						USGS MACS-3						JCp-1					
	Reference value [µg/g]	1SD - reference value [µg/g]	Reference	Mean analytical results [µg/g]	1SD - mean analytical results [µg/g]	Difference from reference value [%]	Reference value [µg/g]	1σ - reference value [µg/g]	Reference	Mean analytical results [µg/g]	1SD - mean analytical results [µg/g]	Difference from reference value [%]	Reference value [µg/g]	1σ - reference value [µg/g]	Reference	Mean analytical results [µg/g]	1SD - mean analytical results [µg/g]	Difference from reference value [%]
B	6	1	*	6.23	0.39	3.9	8.2	1.1	*	11.7	1.0	42.3	48.7	1.8	**	55.4	2.7	13.8
Sr	342	4	*	337	3	-1.6	6640	170	*	7180	135	8.1	7216	288	**	7695	123	6.6

* GeoReM database (version 27) preferred values

** GeoReM database (version 27) mean of published values

Supplementary References

1. McCulloch, M. T., D'Olivo, J. P., Falter, J., Holcomb, M. & Trotter, J. A. Coral calcification in a changing World and the interactive dynamics of pH and DIC upregulation. *Nat. Commun.* **8**, 15686 (2017).
2. D'Olivo, J. P., Sinclair, D. J., Rankenburg, K. & McCulloch, M. T. A universal multi-trace element calibration for reconstructing sea surface temperatures from long-lived *Porites* corals: Removing 'vital-effects'. *Geochim. Cosmochim. Acta* **239**, 109–135 (2018).
3. Thompson, D. et al. Marginal reefs under stress: physiological limits render Galápagos corals susceptible to ocean acidification and thermal stress. *AGU Advances* **3**, e2021AV000509 (2022).
4. Canesi, M. et al. Differences in carbonate chemistry up-regulation of long-lived reef building corals. *Sci. Rep.* **13**, 11589 (2023).
5. Canesi, M. et al. Sea surface temperature reconstruction in the Pacific Ocean using multi-elemental proxy in *Porites* and *Diploastrea* corals: Application to Palau Archipelago. *Chem. Geol.* **645**, 121884 (2024).
