# Supplementary material for: Mid-Miocene warmth pushed fossil coral calcification to physiological limits in high-latitude reefs
Source: Commun Earth Environ. 2025 Jul 19;6(1):569. doi: 10.1038/s43247-025-02559-9 (PMC12274133; doi:10.1038/s43247-025-02559-9)
Supplement: Supplementary file 3 — Description of Additional Supplementary Files [file 43247_2025_2559_MOESM3_ESM.pdf]

Description of Additional Supplementary Files

File name: Supplementary Data

Description: Source data for Figures 2 and 3, and Supplementary Figures 2 (mid-Miocene *Porites*), 3, 4 and 5.
