# Supplementary material for: Mid-Miocene warmth pushed fossil coral calcification to physiological limits in high-latitude reefs
Source: Commun Earth Environ. 2025 Jul 19;6(1):569. doi: 10.1038/s43247-025-02559-9 (PMC12274133; doi:10.1038/s43247-025-02559-9)
Supplement: Supplementary file 5 — Reporting Summary [file 43247_2025_2559_MOESM5_ESM.pdf]

nature portfolio

Corresponding author(s): Markus Reuter

Last updated by author(s): 2025/06/03

Reporting Summary

Nature Portfolio wishes to improve the reproducibility of the work that we publish. This form provides structure for consistency and transparency in reporting. For further information on Nature Portfolio policies, see our Editorial Policies and the Editorial Policy Checklist.

Statistics

For all statistical analyses, confirm that the following items are present in the figure legend, table legend, main text, or Methods section.

n/a	Confirmed	
☐	☒	The exact sample size (*n*) for each experimental group/condition, given as a discrete number and unit of measurement
☐	☒	A statement on whether measurements were taken from distinct samples or whether the same sample was measured repeatedly
☒	☐	The statistical test(s) used AND whether they are one- or two-sided<br>*Only common tests should be described solely by name; describe more complex techniques in the Methods section.*
☒	☐	A description of all covariates tested
☒	☐	A description of any assumptions or corrections, such as tests of normality and adjustment for multiple comparisons
☐	☒	A full description of the statistical parameters including central tendency (e.g. means) or other basic estimates (e.g. regression coefficient) AND variation (e.g. standard deviation) or associated estimates of uncertainty (e.g. confidence intervals)
☒	☐	For null hypothesis testing, the test statistic (e.g. *F*, *t*, *r*) with confidence intervals, effect sizes, degrees of freedom and *P* value noted<br>*Give P values as exact values whenever suitable.*
☒	☐	For Bayesian analysis, information on the choice of priors and Markov chain Monte Carlo settings
☒	☐	For hierarchical and complex designs, identification of the appropriate level for tests and full reporting of outcomes
☒	☐	Estimates of effect sizes (e.g. Cohen's *d*, Pearson's *r*), indicating how they were calculated

Our web collection on statistics for biologists contains articles on many of the points above.

Software and code

Policy information about availability of computer code

Data collection	The free available X-ray densitometry app CoralXDS (2022) was used to measure skeletal density.
Data analysis	Microsoft Office Excel, Golden Software Grapher 23

For manuscripts utilizing custom algorithms or software that are central to the research but not yet described in published literature, software must be made available to editors and reviewers. We strongly encourage code deposition in a community repository (e.g. GitHub). See the Nature Portfolio guidelines for submitting code & software for further information.

Data

Policy information about availability of data

All manuscripts must include a data availability statement. This statement should provide the following information, where applicable:

- Accession codes, unique identifiers, or web links for publicly available datasets
- A description of any restrictions on data availability
- For clinical datasets or third party data, please ensure that the statement adheres to our policy

All data generated for this study are included in this published article and its Supplementary Data file.

Research involving human participants, their data, or biological material

Policy information about studies with human participants or human data. See also policy information about sex, gender (identity/presentation), and sexual orientation and race, ethnicity and racism.

Reporting on sex and gender	The study does not involve human participants, their data or biological material.
Reporting on race, ethnicity, or other socially relevant groupings	*Please specify the socially constructed or socially relevant categorization variable(s) used in your manuscript and explain why they were used. Please note that such variables should not be used as proxies for other socially constructed/relevant variables (for example, race or ethnicity should not be used as a proxy for socioeconomic status).* *Provide clear definitions of the relevant terms used, how they were provided (by the participants/respondents, the researchers, or third parties), and the method(s) used to classify people into the different categories (e.g. self-report, census or administrative data, social media data, etc.)* *Please provide details about how you controlled for confounding variables in your analyses.*
Population characteristics	*Describe the covariate-relevant population characteristics of the human research participants (e.g. age, genotypic information, past and current diagnosis and treatment categories). If you filled out the behavioural & social sciences study design questions and have nothing to add here, write "See above."*
Recruitment	*Describe how participants were recruited. Outline any potential self-selection bias or other biases that may be present and how these are likely to impact results.*
Ethics oversight	*Identify the organization(s) that approved the study protocol.*

Note that full information on the approval of the study protocol must also be provided in the manuscript.

Field-specific reporting

Please select the one below that is the best fit for your research. If you are not sure, read the appropriate sections before making your selection.

☐ Life sciences ☐ Behavioural & social sciences ☒ Ecological, evolutionary & environmental sciences

For a reference copy of the document with all sections, see nature.com/documents/nr-reporting-summary-flat.pdf

Ecological, evolutionary & environmental sciences study design

All studies must disclose on these points even when the disclosure is negative.

Study description	X-ray densitometry and geochemical analysis of fossil corals from the ancient Central Paratethys Sea to assess coral resilience to ocean warming and acidification during the Middle Miocene.
Research sample	Three fragments of fossil Porites corals.
Sampling strategy	Since fossil corals that have retained their original skeletal mineralogy and porosity, which is essential for our analyses, are extremely rare, the sample size is limited to three specimens.
Data collection	Skeletal bulk density was measured on the digital X-ray images of the coral slabs along transects parallel to the corallites along the main growth at the Institute for Earth System Science and Remote Sensing, Leipzig University (P. Spreter) using the X-ray densitometry app CoralXDS. Trace element concentrations were determined at the Institute of Geosciences, Johannes Gutenberg University Mainz, Germany (P. Spreter, R. Mertz-Kraus) with an Agilent 7500ce inductively coupled plasma-mass spectrometer (ICP-MS) coupled to an ESI NWR193 ArF excimer laser ablation (LA) system equipped with a TwoVol2 ablation cell. The isotopic analyses were conducted at the Institute for Earth System Science and Remote Sensing, Leipzig University, Germany (P. Spreter) using a Kiel IV online carbonate preparation line connected to a MAT 253 isotope ratio mass spectrometer.
Timing and spatial scale	n/a
Data exclusions	No data were excluded from the analyses.
Reproducibility	We used standardized methods and detailed methodology documentation to enhance reproducibility. However, due to the limited sample availability we cannot test if our results generalize to larger populations.
Randomization	The samples were allocated to the study group according to genus, state of preservation, palaeogeographic setting and geological age. Randomization was not possible due to the limited availability of fossil material that can be used for this study.
Blinding	Blinding was not possible due to the small sample size.

Did the study involve field work? ☐ Yes ☒ No

Reporting for specific materials, systems and methods

We require information from authors about some types of materials, experimental systems and methods used in many studies. Here, indicate whether each material, system or method listed is relevant to your study. If you are not sure if a list item applies to your research, read the appropriate section before selecting a response.

Materials & experimental systems

n/a	Involved in the study
☒	☐ Antibodies
☒	☐ Eukaryotic cell lines
☐	☒ Palaeontology and archaeology
☒	☐ Animals and other organisms
☒	☐ Clinical data
☒	☐ Dual use research of concern
☒	☐ Plants

Methods

n/a	Involved in the study
☒	☐ ChIP-seq
☒	☐ Flow cytometry
☒	☐ MRI-based neuroimaging

Palaeontology and Archaeology

Specimen provenance: The Dras and Poetz corals come from the collection of the Natural History Museum Vienna (Austria) and were collected at the beginning of the last century. Sample U4 was provided by Nàdai László from Hungary.

Specimen deposition: Samples Dras and Poetz are deposited in the Natural History Museum Vienna (Austria) with collection numbers NHMW 2024/0194/0001 and NHMW 2024/0194/0002. U4 is deposited in the collection of the Institute of Geography and Geology of Greifswald University (Germany) with collection number GG 520.

Dating methods: No new dating was carried out.

☐ Tick this box to confirm that the raw and calibrated dates are available in the paper or in Supplementary Information.

Ethics oversight: As the study is based entirely on material from old collections that does not originate from former colonial territories or endangered species, ethical approval or guidance of the study protocol is not required.

Note that full information on the approval of the study protocol must also be provided in the manuscript.

Plants

Seed stocks: No plant material was involved in this study.

Novel plant genotypes: *Describe the methods by which all novel plant genotypes were produced. This includes those generated by transgenic approaches, gene editing, chemical/radiation-based mutagenesis and hybridization. For transgenic lines, describe the transformation method, the number of independent lines analyzed and the generation upon which experiments were performed. For gene-edited lines, describe the editor used, the endogenous sequence targeted for editing, the targeting guide RNA sequence (if applicable) and how the editor was applied.*

Authentication: *Describe any authentication procedures for each seed stock used or novel genotype generated. Describe any experiments used to assess the effect of a mutation and, where applicable, how potential secondary effects (e.g. second site T-DNA insertions, mosiacism, off-target gene editing) were examined.*
